# Curious Case of Volitional Control over Involuntary Acts

**Published:** 1881-01

**Authors:** 


Curious Cases of Volitional Control over Involuntary Acts.—Dr. Berliakoff reports in *Vratch*, 1880, No. 8, a case of a man suffering from syphilitic ulceration of the rectum, who succeeded in gaining such control over his sphincters, that he could under certain conditions, open the anal orifice, permitting a perfect inspection of the rectal mucous membrane to the depth of seven centimetres, the anus appearing as a hole two centimetres in diameter. He could retain the parts in this condition for ten to fifteen minutes, becoming very much fatigued at the end. To accomplish it, he had to place himself in the position known as *a la vache*, cover his head with a blanket or a gown, stop breathing and draw in the abdomen; perfect silence was absolutely necessary. He could not give any explanation of how he was doing this. Silence and darkness were necessary to permit him to concentrate his mind on the act. He was led to this by a desire to assist his physician, as local manipulations were very difficult and painful. Prof. Manassein, editor of *Vratch*, speaks of two medical students; one of whom can accelerate and the other retard the pulse-rate at will.—*Western Lancet.*

